# Comparison of Plant Metabolites in Root Exudates of *Lolium perenne* Infected with Different Strains of the Fungal Endophyte *Epichloë festucae* var. *lolii*

**DOI:** 10.3390/jof7020148

**Published:** 2021-02-18

**Authors:** Aurora Patchett, Jonathan A. Newman

**Affiliations:** 1 Department of Earth Sciences, University of Gothenburg, 405 30 Gothenburg, Sweden; aurora.patchett@gu.se; 2 Department of Biology, Wilfrid Laurier University, Waterloo, ON N2L 3C5, Canada

**Keywords:** *Epichloë festucae* var. *lolii*, *Lolium perenne*, endophyte strains, metabolomics

## Abstract

*Lolium perenne* infected with the fungal endophyte *Epichloë festucae* var. *lolii* have specific, endophyte strain-dependent, chemical phenotypes in their above-ground tissues. Differences in these chemical phenotypes have been largely associated with classes of fungal-derived alkaloids which protect the plant against many insect pests. However, the use of new methodologies, such as various omic techniques, has demonstrated that many other chemical changes occur in both primary and secondary metabolites. Few studies have investigated changes in plant metabolites exiting the plant in the form of root exudates. As root exudates play an essential role in the acquisition of nutrients, microbial associations, and defense in the below-ground environment, it is of interest to understand how plant root exudate chemistry is influenced by the presence of strains of a fungal endophyte. In this study, we tested the influence of four strains of *E. festucae* var. *lolii* (E+ (also known as Lp19), AR1, AR37, NEA2), and uninfected controls (E−), on *L. perenne* growth and the composition of root exudate metabolites. Root exudates present in the hydroponic water were assessed by untargeted metabolomics using Accurate-Mass Quadrupole Time-of-Flight (Q–TOF) liquid chromatography–mass spectrometry (LC–MS). The NEA2 endophyte strain resulted in the greatest plant biomass and the lowest endophyte concentration. We found 84 metabolites that were differentially expressed in at least one of the endophyte treatments compared to E− plants. Two compounds were strongly associated with one endophyte treatment, one in AR37 (*m*/*z* 135.0546 RT 1.17), and one in E+ (*m*/*z* 517.1987 RT 9.26). These results provide evidence for important changes in *L. perenne* physiology in the presence of different fungal endophyte strains. Further research should aim to connect changes in root exudate chemical composition with soil ecosystem processes.

## 1. Introduction

Fungal endophytes of the genus *Epichloë* can be found in many species of cool season grass [1]. *Lolium perenne* is one such grass that commonly forms an association with *Epichloë festucae* var. *lolii* (≡*Neotyphodium lolii*, ≡*Acremonium lolii*; [2]). This symbiotic grass–fungal endophyte relationship is most likely a mutualism [3]: the endophyte benefits from the nutrients and the protection which the host provides, while the grass receives several fitness benefits such as improved growth in terms of biomass production, drought tolerance, nutrient acquisition, and deterrence or toxicity to herbivores [4,5,6]. Due to the intimate nature of the plant–fungal endophyte relationship, their metabolomes are inextricably linked. Therefore, it is only possible to consider the plant–fungal metabolome as a whole. Many changes in the plant-endophyte metabolome may contribute to the fitness of the grass host [7,8,9]. Both transcriptomic and metabolomic analyses have demonstrated that *L. perenne* infection by *E. festucae* results in changes in primary and secondary metabolism, as well as stress-related gene expression [9]. These changes in the host plant’s chemistry have ecological consequences both above- and below-ground.

Metabolomics is an emerging technique in the toolbox of plant ecologists, several of whom have utilized it for analyses of shoot tissue, guttation fluid, apoplastic fluid [7,10,11,12,13], and to a lesser extent root tissue and root exudate of infected hosts [14,15,16]. These metabolomic techniques have not only led to the discovery of new metabolites, such as alkaloids, extracellular siderophores, and cyclic oligopeptides, they have also provided insight into changes in primary and secondary metabolism in the plant–fungal endophyte metabolome [7,11,17].

Fundamental changes observed in primary metabolism include increases in non-structural carbohydrates (water soluble carbohydrates (WSCs), sugar alcohols, and storage carbohydrates) and decreases in nitrogenous compounds [9,10,11,18,19,20]. In perennial ryegrass (cv. Samson), the leaf and pseudostems of endophyte-infected plants have higher concentrations of fructans, a primary storage carbohydrate, and glucose, fructose, and sucrose, involved in carbohydrate metabolism, and a lower concentration of starch and soluble protein [19]. Non-structural carbohydrates and nitrogen are essential to both plant and endophyte for growth, development, and signaling [21,22,23]. Changes in the C:N ratio in infected plants may be a result of a metabolic cost associated with hosting the endophyte, wherein the fungal endophyte has a higher nitrogen demand [10,20,24]. These fungal endophytes can use the plant host’s resources to synthesize various nitrogen-rich bioactive secondary metabolites, such as alkaloids. The concentration of alkaloids present in plant tissue is linearly related to the concentration of fungal endophyte in plant tissue [11,25,26,27,28]. The most studied alkaloids in grass–fungal endophyte associations include pyrrolopyrazines (peramine; [29]), indole diterpenes (lolitrem B; [30]), and lysergyls (ergovaline; [31]), all produced by the ‘common toxic’ (E+) strain of the endophyte. Natural variants of *Epichloë* fungi exist, which differ in their alkaloid profiles [32]. These variants are often referred to as ‘novel’ or ‘selected’ endophytes—terms used in the grass breeding industry. The novel endophyte strain AR37 produces a unique group of epoxy-janthitrems [33]. These fungal-derived alkaloids play a major role in plant host resistance to invertebrate herbivores [34,35]. Other metabolites that play a role in plant host resistance are phenolic and volatile organic compounds [36,37,38,39], and the plant hormones jasmonic and salicylic acids [40,41] all of which may change in composition and quality in fungal endophyte-infected host tissue.

The majority of research investigating the interactions, influences, and consequences of plant–fungal endophyte relationships has focused on above-ground effects. Research investigating the below-ground impacts of these relationships is still limited. Below-ground ecosystem processes, such as decomposition and nutrient cycling, are influenced by interacting plants and soil organisms. These processes are essential to the transfer of resources throughout the foodweb. The bulk of these interactions occur in the rhizosphere, the root surface and soil interface, and are heavily influenced by the quality and quantity of plant outputs via the roots (i.e., root exudation). Therefore, it is important to improve our understanding of the below-ground component of plant–fungal endophyte relationships to determine their role in grassland ecosystems.

Most of plant–fungal endophyte metabolome research has focused on above-ground tissues; far less is known about root and root exudate chemistry. Those studies investigating root and root exudate chemistry have predominantly focused on a related grass–fungal endophyte association between *Schedonorus arundinaceus* (tall fescue) and *Epichloë coenophiala* (≡*Neotyphodium coenophialum*, ≡*Acremonium coenophialum*). As in above-ground tissues, fungal endophytes can induce changes in root exudate composition and quantity [14,42]. There are increases in soluble organic carbon and carbohydrates [42]. Guo et al. [14] found that E+ infected plants had more total carbon and phenolic compounds in their root exudates than plants infected with novel endophytes, which were also different from each other. They also found that amines, growth factors, and vitamins were affected by endophyte status. In *L. perenne*, Ren et al. [20] found that soluble sugars increased in the roots of endophyte-infected plants, but this was only observed at high nitrogen levels (see also [43]). Wakelin et al. [16] extracted metabolites from the rhizosphere of *L. perenne* plants infected with selected strains of *E. festucae* var. *lolii* and found that metabolite profiles between E+ and E− plants differed most in the alkane hydrocarbon derivatives (i.e., lipids).

There is minimal information available on the root exudate composition of *L. perenne* infected with different strains of *E. festucae* var. *lolii*. Therefore, our primary purpose was to improve our understanding of how the root exudates in perennial ryegrass differ, with and without endophyte infection, and how the different strains of the endophyte alter root exudate composition. A hydroponic growth system, while limited in ecological relevance, was chosen to facilitate detection and quantification of the changes between the plant and plant–fungal endophyte metabolome. Plant metabolites are diverse and complex; no one analytical method will capture the whole plant–fungal metabolome. In this study, we conducted untargeted metabolomics using Accurate-Mass Quadrupole Time-of-Flight (Q-TOF) LC–MS to detect polar compounds. We hypothesized that endophyte presence and endophyte concentration would have strain-specific effects on plant growth and root exudate composition.

We asked, and tentatively answered, the following questions:

Q1: Does plant ‘performance’ (root dry mass (DM), shoot DM, root to shoot ratio) differ between infected and uninfected plants?
Q2: Does plant ‘performance’ differ between plants infected with different endophyte strains?
Q3: Do the *in planta* endophyte concentrations differ between endophyte strains?
Q4: Do the exudate metabolomes differ between infected and uninfected plants?
Q5: Do the exudate metabolomes differ between plants infected with different strains?
Q6: Do relative abundances of any of the exudate metabolites correlate with endophyte concentration?

## 2. Methods

To isolate the effects of endophyte infection on perennial ryegrass plant metabolites in root exudates, we designed a hydroponic experiment to collect root exudate to identify individual metabolites and to capture its metabolic profile. We also estimated the concentration of endophyte infection to determine if it was a contributing factor in the metabolic profile. Additional measurements were taken to determine the effect of endophytes on plant growth.

### 2.1. Experimental Design

The experiment was performed in a glasshouse from January to April 2016. The temperature was maintained at ∼23 °C and supplementary lighting was provided on a 16:8 h light:dark cycle. The hydroponic system comprised 90 pots set up as a randomized block design (9 blocks × 5 endophyte treatments with 2 replicates of each endophyte treatment per block).

### 2.2. Sample Preparation

*Lolium perenne* seeds (cultivar Alto), either uninfected (E−) or infected with one of four endophyte strains (AR1, AR37, NEA2, E+ (also known as Lp19)), were stored at −20 °C. Endophyte status of seeds was previously confirmed using an immunoblot assay (Phytoscreen seed endophyte detection kit; Agrinostics Ltd. Co., Watkinsville, GA, USA). We assumed that the genetic variation among seeds was randomly distributed among the experimental groups. Single seeds were sown into Rockwool^™^(Rockwool International A/S, Hedehusene, Denmark) and grown for nine weeks in the glasshouse. Whole plants in Rockwool^™^ were then transferred to the hydroponic system comprising individual 1 L silver clay pots (ceramic, inert), filled to the top with fertilizer water (Plant Products^®^, 20-8-20 All Purpose High Nitrate, 1.25 g per liter, 250 parts nitrogen, pH adjusted to 6.0). Fertilizer water was replenished every other day. Pots were aerated through a plastic tube that pumped in a steady flow. Plants were acclimated and grown for an additional four weeks At the end of this growth period, the pot’s fertilizer water was discarded and replaced with deionized water to sample root exudate.

### 2.3. Sample Collection

After 24 h in aerated non-circulating deionized water, all plants and root exudate were harvested. Two individual tillers (consisting of stem, sheath, and blade tissue) and a sample of the root tissue from each plant were removed and preserved in liquid nitrogen. The tiller and root tissue were then freeze-dried and stored at −20 °C for further molecular analyses. From one tiller, the stem and sheath tissue were separated from the blade for use in qPCR. All freeze-dried samples of plant tissue (tiller and root) were weighed and then ground using a 2010 Geno/Grinder^®^ (SPEX^®^ SamplePrep, Metuchen, NJ, USA) tissue homogenizer. Ground plant tissue was stored at −20 °C for immediate use.

All remaining plant tissue (roots and shoots) was harvested by cutting the shoot and root portions as closely as possible to the Rockwool^™^ medium. We weighed oven dried plant tissue to determine overall plant biomass. Lastly, 50 mL of the deionized water from the hydroponic pots was collected and placed in falcon tubes (Fisherbrand), vacuum filtered through nylon 0.45 μm filters (Merck Millipore Ltd., Oakville, ON, Canada), and stored in 10 mL Falcon tubes at −80 °C.

### 2.4. Endophyte Concentration

Endophyte concentration in plant tissue was measured by quantitative polymerase chain reaction (qPCR) following methods as described in Ryan et al. [44,45]. Briefly, genomic DNA (gDNA) was extracted from 20 mg of ground stem and sheath tissue, using DNeasy Plant Mini Kit (Qiagen Inc., Toronto, ON, Canada) in conjunction with the QIAcube^®^ (Qiagen Inc., Toronto, ON, Canada) as per manufacturer’s instructions. Total gDNA (plant and fungal) was measured on a NanoDrop^®^ 2000, and diluted to a working concentration of 0.5 ng total gDNA/μL using Millipore water. PCR reactions of 15 μL were prepared with 3 ng of gDNA, 0.5 μmol of each endophyte primer (Translation elongation factor 1-α: *tef*A–F $5^{\prime }$-CACGTACTGACTGAAGCGTAGC-$3^{\prime }$; *tef*A–R $5^{\prime }$–CAATGCAGCGAGTGAACATC-$3^{\prime }$), and 1× concentration of LightCycler^®^ 480 SYBR Green I Master (Roche, Canada). The primers were designed for amplification of the *tef*A region in *N. coenophialum* as described in Ryan et al. [44,45]. The sequencing record of *E. festucae* var. *lolii* indicated that the region in the *tef*A gene was the same for both species. We assume that the targeted region is identical for all endophyte strains used. Samples were quantified in triplicate. Both dilutions and plating were carried out by an automated PCR set-up instrument, QIAgility^®^ (Qiagen Inc., Toronto, ON, Canada). PCR reactions were performed on a LightCycler^®^ 480 Instrument II (Roche, Laval, QC, Canada). The PCR thermocycling conditions were as follows: initial denaturation for one cycle at 95 °C for 5 min, followed by amplification for 45 cycles of 95 °C for 10 s, 64 °C for 10 s, and 72 °C for 10 s. The Tm of the PCR product was 83.75 ± 0.25, as determined by standard protocols on the Lightcycler. One alteration to the quantification method was needed when samples were below the detection limit. In these cases, the samples were re-run using 9 ng of gDNA. If endophyte concentration was still below the detection limit, the sample was considered to have no endophyte infection.

### 2.5. Metabolomic Profile of Root Exudate

Frozen root exudate samples were thawed, and 200 μL of each were transferred into 350 μL glass vials (Thermo Scientific, Mississauga, ON, Canada,^™^ National MS Certified, MSCERT5000-37LVW). No solvent extraction step or concentration step was conducted.

To determine the root exudate composition, liquid chromatography–mass spectrometry (LC–MS) analyses were performed on an Agilent 1200 HPLC liquid chromatograph interfaced with an Agilent UHD 6530 Q-Tof mass spectrometer at the Mass Spectrometry Facility of the Advanced Analysis Centre, University of Guelph. A C18 column (Agilent, Mississauga, ON, Canada, Poroshell 120, EC-C18 50 mm × 3.0 mm 2.7 μm) was used for chromatographic separation with the following solvents: water with 0.1% formic acid (A) and acetonitrile with 0.1 formic acid (B). The mobile phase gradient was as follows: initial conditions were 10% B hold for 1 min then increasing to 100% B in 29 min followed by column wash at 100% B for 5 min and 20 min re-equilibration. The flow rate was maintained at 0.4 mL/min. The mass spectrometer electrospray capillary voltage was maintained at 4.0 kV and the drying gas temperature at 250 °C with a flow rate of 8 L/min. Nebulizer pressure was 206.8 kPa (30 psi) and the fragmentor was set to 160. Nitrogen was used as both nebulizing and drying gas. The mass-to-charge ratio was scanned across the *m*/*z* range of 50–1500 *m*/*z* in 4GHz (extended dynamic range) positive and negative ion modes. The acquisition rate was set at 2 spectra/s. The instrument was externally calibrated with the ESI TuneMix (Agilent, Agilent, Mississauga, ON, Canada). The sample injection volume was 10 μL.

### 2.6. Metabolomic Data Analysis

The mass spectrometry data were further processed using Agilent MassHunter WorkStation software (Agilent, Mississauga, ON, Canada, MassHunter Profinder B.08.00). Recursive molecular feature extraction (rMFE) was used for binning and alignment of molecular features. The rMFE is an algorithm that groups related co-eluting ions (i.e., isotopes, adducts, and dimers) into a single compound and then creates compound chromatograms. The rMFE step also filters out noise and reduces false positives. Molecular features were aligned based on a retention time window of 0.40 min and a mass window of 40.00 ppm + 2.00 mDa, and an absolute height of at least 3000 counts. Aligned features found in at least six replicates in one treatment group (n=18) were retained. Molecular features were extracted as compound exchange format (cef) files and imported into Agilent’s Mass Profiler Professional (MPP) software (version B14.5). We used MPP for statistics visualization, and annotation and identification of compounds. In MPP, compounds that were different and/or unique to certain treatment groups were noted. To aid in improving compound identification, a subset of samples with the highest intensities of compounds was re-run using MS/MS.

### 2.7. Statistical Analyses

We follow Wasserstein et al. [46] in reporting exact *p*-values and avoiding the use of the terms “significant” and “non-significant.” Furthermore, we follow Greenland [47] by also reporting the Shannon information transformation, $s=-\log_{2}\left(P\right)$. As Greenland notes, larger values of *s* correspond to more evidence against the null hypothesis. The Shannon information transformation can be understood by analogy to a test of “fairness” for a coin, such that *s* is the number of consecutive coin tosses that result in heads. Thus, if p=0.04, then this is equivalent to the same amount of information against the null hypothesis of fairness as obtaining $-\log_{2}\left(0.04\right)=4.64$ heads in a row ([46], p. 12).

#### 2.7.1. Contrast Analysis to Compare Uninfected and Infected Plants

To compare uninfected to infected plants, we used a contrast analysis. The first step was to conduct an ANOVA to remove the variation due to the blocks [48]. The model is shown below:

(1) $$\begin{array}{cc} Y_{i,j} & =\mu +\beta_{i}+\tau_{j}+\epsilon_{i,j},\\ \mathrm{for}\;i & ={\mathrm{block}}_{\left(1\right)},\ldots ,{\mathrm{block}}_{\left(9\right)},\\ \mathrm{and}\;j & =E-,E+,\mathrm{AR}1,\mathrm{AR}37,\mathrm{NEA}2, \end{array}$$

where $Y_{i,j}$ is the value of the dependent variable for a plant in the *i*th block and the *j*th strain, $\beta_{i}$ is the effect of the *i*th block, $\tau_{j}$ is the effect of the *j*th endophyte strain, and $\epsilon_{i,j}$ is the random error.

Following the ANOVA, we conducted a pre-planned contrast to compare the mean of the endophyte-free plants to the mean of the endophyte-infected plants. Formally, we test the null hypothesis that:

(2) H0:X¯E−−(14)(X¯E++X¯AR1+X¯AR37+X¯NEA2)=0,

where the ${\bar{X}}_{i}$ denotes the arithmetic means of the different endophyte treatments.

#### 2.7.2. ANOVA and Tukey’s HSD to Compare Effects of Different Endophyte Strains

To compare the effects of different endophyte strains, we first excluded the E− plants. For the remaining infected plants, we conducted an ANOVA followed by Tukey’s Honestly Significant Difference test [49].

(3) $$\begin{array}{cc} Y_{i,j} & =\mu +\beta_{i}+\tau_{j}+\epsilon_{i,j},\\ \mathrm{for}\;i & ={\mathrm{block}}_{\left(1\right)},\ldots ,{\mathrm{block}}_{\left(9\right)},\\ \mathrm{and}\;j & =E+,\mathrm{AR}1,\mathrm{AR}37,\mathrm{NEA}2. \end{array}$$

A large value of *s* (derived from the *F*-ratio and *p*-value for the strain effect) indicates that there are differences amongst strains and the Tukey’s HSD test helps us identify the source of those differences.

#### 2.7.3. Analysis of Plant Performance and Endophyte Concentration

For **question 1**, we used the contrast analysis shown in Section 2.7.1 to address this question. One sample in the NEA2 treatment group had an extreme ‘outlier’. We ran the model with and without the outlier and found that the results were not qualitatively different. The results presented below include the outlier since we have no objective reason to exclude it. We analyzed the Box-Cox transformed [50] data to meet the assumptions of ANOVA. However, for ease of interpretation, we present the untransformed means and standard errors in the results section. Note that only two of the three dry matter variables are independent of each other (because total DM = shoot DM + root DM).

For **question 2**, we used the analysis shown in Section 2.7.2. Again, we analyzed the Box-Cox transformed data but present the untransformed means and standard errors in the results section. We used a Tukey’s Honestly Significant Difference (HSD) test to compare each strain with the others. The *s*-value and Tukey’s HSD test directly address question 2.

For **question 3**, we analyzed the endophyte concentration (gene copies ng${}^{-1}$ of total gDNA) using the analysis shown in Section 2.7.2. Again, we analyzed the Box-Cox transformed data but present the untransformed means and standard errors in the results section; and again, we used Tukey’s HSD test to compare each endophyte strain to each other, directly addressing question 3.

#### 2.7.4. Analysis of the Exudate Metabolome

Metabolite data, collected in positive and negative ion modes, were subjected to statistical and visual differential analysis in MPP separately. We applied the Benjamini–Hochberg false discovery rate (FDR) correction [51] to control for the number of false positives, resulting from multiple testing of *p*-values (p<0.05). We also applied an additional fold-change filter ≥2.0 to identify important metabolites.

For all of the metabolites that passed both the FDR correction and the fold-change filter, we used principal component analysis (PCA) and hierarchical cluster analysis (HCA) to visualize the data. Hierarchical clustering was used to group compounds showing large differences in abundance (as determined by an ANOVA and fold-change) in clusters by metabolite and by endophyte treatment using a Euclidean distance metric [52,53] and Ward’s Linkage rule [54].

For **question 4**, we retained the first three principal components from the PCA for each of the ion modes. We analyzed these principal components using the contrast analysis shown in Section 2.7.1. This contrast directly tests question 4.

For **question 5**, we re-ran the PCA this time excluding the E− plants, and subjected the first three principal components to the analysis shown in Section 2.7.2 to examine the differences between the endophyte strains.

Finally, for **question 6**, we used the relative abundance data for each of the metabolites that passed the FDR correction and the fold-change filter, and the endophyte concentrations (gene copies ng${}^{-1}$ gDNA) to calculate Spearman’s Rank Correlation (ρ). We examined ρ for the correlation between endophyte concentration and each of the relative abundances of each metabolite in both the negative and positive ion modes. For the correlations in each ion mode, we then applied an FDR correction as described above. These correlations directly address question 6.

## 3. Results

### 3.1. Does Plant ‘Performance’ Differ between Infected and Uninfected Plants?

**Figure 1:**
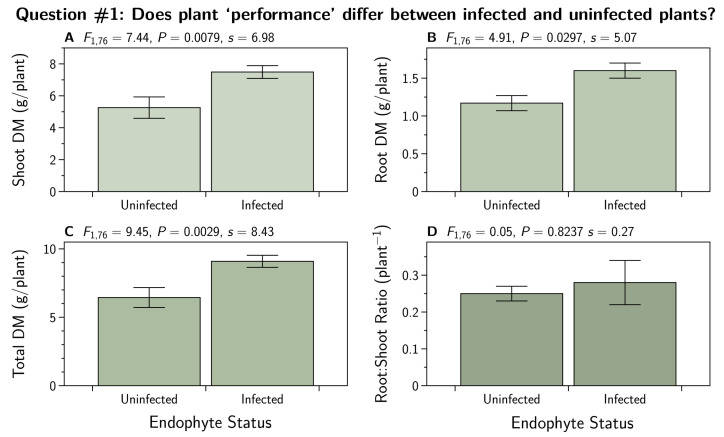
Dry matter (DM) biomass for shoots (**A**), roots (**B**), and total plant (**C**) of *Lolium perenne* either *uninfected* (E−), or *infected* with different strains of *Epichloë festucae* var. *lolii* endophyte (common toxic strain, E+; and novel strains AR1, AR37, and NEA2). Each treatment n=18. Additinally shown is the root to shoot ratio (**D**).

We subjected root, shoot, and total biomass DM, as well as the root to shoot ratios, to the contrast analysis (Section 2.7.1) comparing the mean of the uninfected plants to the mean of the infected plants. The results are shown in Figure 1. There were convincing differences seen in all three biomass DM measures while the root to shoot ratios were about equal between the two groups.

### 3.2. Does Plant ‘Performance’ Differ between Plants Infected with Different Endophyte Strains?

**Figure 2:**
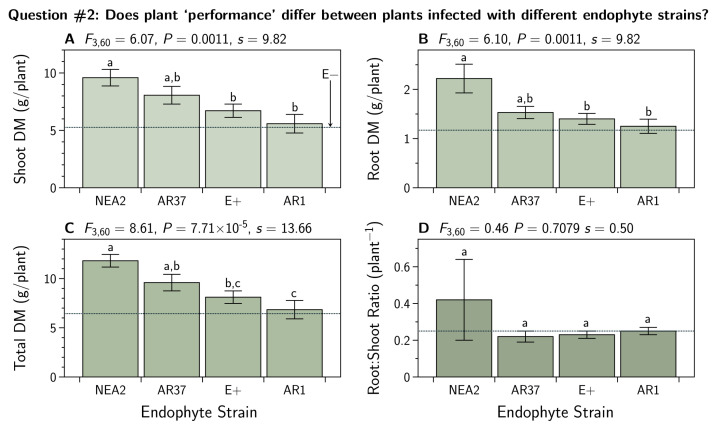
Dry matter (DM) biomass for shoots (**A**), roots (**B**), and total biomass (**C**) of *Lolium perenne* infected with different strains of *Epichloë festucae* var. *lolii* endophyte (common toxic strain, E+; and novel strains AR1, AR37, and NEA2). Each treatment n=18. Means with the same letter do not differ (Tukey HSD test, p>0.05). The dashed horizontal line indicates the treatment mean for the E− plants that were excluded from this analysis (see Figure 1). Additionally shown are the root to shoot ratios (**D**).

Excluding the uninfected plants, we subjected root, shoot, and total biomass DM, as well as the root to shoot ratios, to an ANOVA and subsequent Tukey’s HSD test (Section 2.7.2). The results are shown in Figure 2. There we see large variation among the means for each endophyte strain for all three biomass DM measures, but not for the root to shoot ratio.

### 3.3. Do the in Planta Endophyte Concentrations Differ between Endophyte Strains?

**Figure 3:**
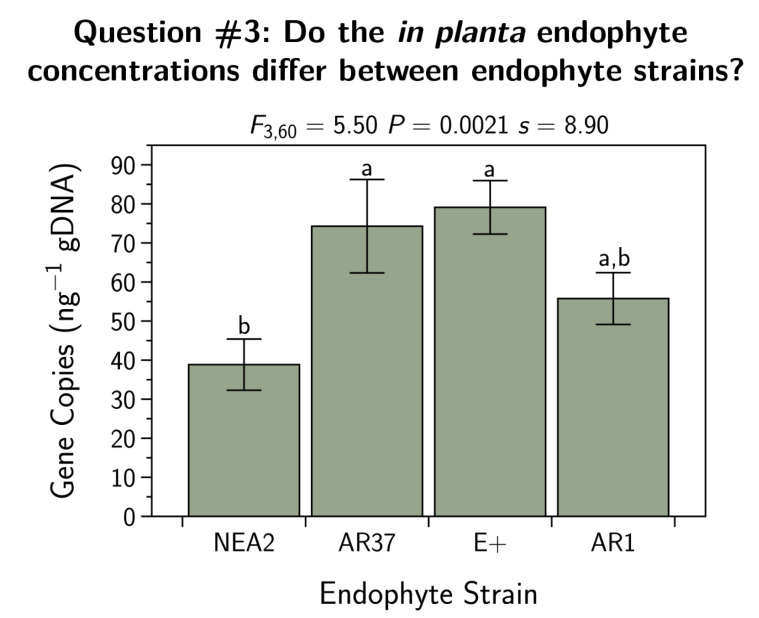
Fungal endophyte concentrations for *Lolium perenne* plants infected with different strains of *Epichloë festucae* var. *lolii* endophyte. Sample sizes differed between the treatments (E+, n=18; AR1, n=17; AR37, n=17; NEA2, n=15). Shown are the means ± the SEM. Means with the same letter do not differ (Tukey HSD test, p>0.05).

We subjected the endophyte concentration in the plant sheath tissue (gene copies ng${}^{-1}$ gDNA) to an ANOVA and subsequent Tukey’s HSD test (Section 2.7.2). The results are shown in Figure 3. There we can see that there were differences between the strains, with NEA2 having the lowest endophyte concentrations while AR37 and E+ had the highest concentrations.

### 3.4. Do the Exudate Metabolomes Differ between Infected and Uninfected Plants?

We performed an accurate-mass Q-TOF LC/MS-based analysis of metabolites in root exudates. Our goal was to obtain a general overview of metabolomic similarities and differences in plant root exudates between uninfected and infected plants. Feature extraction from the raw data found 62 features from the positive ion mode, and 115 features from the negative ion mode (data not shown).

#### 3.4.1. Positive Ion Mode

An ANOVA showed that 41 of the 62 entities were different at a corrected *p*-value (FDR; [51]) cut-off of 0.05. A subsequent Tukey HSD showed where the differences lay (see Appendix A, Table A1). We applied a fold-change filter to the 41 compounds with differences. Table A2 lists the 24 metabolites found to be differentially expressed in at least one of the endophyte treatments, with p<0.05 and fold-change ≥2.0. Perhaps not surprisingly, the main differences were found in the up and downregulation of the compounds, rather than the presence or absence of compounds. Overall, 12.5% of all metabolites were upregulated in all endophyte treatments when compared to E− treatments. There were no metabolites that were downregulated in all endophyte treatments.

**Figure 4:**
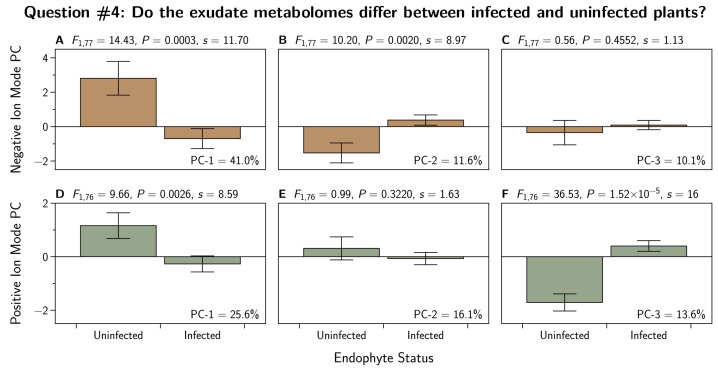
Principal component analysis (PCA) performed on the positive ion mode metabolomic profiles of *Lolium perenne* root exudates infected with strains of *Epichloë festucae* var. *lolii* endophyte. This PCA is based on 60 metabolites in the negative ionization mode (**A**–**C**), and on 24 metabolites in the positive ionization mode (**D**–**F**), found to be differentially-expressed in at least one of the endophyte treatments, with p<0.05 and fold-change >2.0. Each treatment n=18. Plotted are the means and standard errors of the means. Additionally shown are contrast analyses and the corresponding Shannon transformation indices (*s*). Percentages in each subfigure represent the percentage of the total variation in the metabolite set that is explained by each PC.

To address question 4, we first reduced the dimensionality of the 24 metabolites by conducting a principal component analysis. We were able to reduce these 24 variables to three principal components (PC) that together explained more than half of the total variation in the 24 metabolites. The PC-loadings are shown in Table A3. We subjected these three PCs to the contrast analysis described in Section 2.7.1. The results are shown in Figure 4. There we see differences in PC-1 and PC-3 between infected and uninfected plants. There was not convincing evidence to reject the null hypothesis for PC-2.

**Figure 5:**
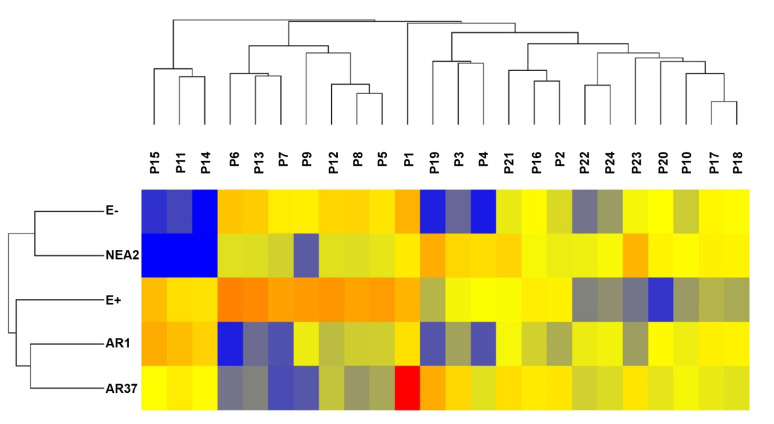
Unsupervised hierarchical clustering of *Lolium perenne* root exudate metabolites, obtained by LC–MS in the positive ion mode. These are the 24 metabolites found to be differentially-expressed in at least one of the endophyte treatments, with p<0.05 and fold-change >2.0. Metabolites are grouped by fungal endophyte treatments based on similar peak intensity profiles. Colors represent normalized intensity values (yellow is the center, blue represents low intensity, red represents high intensity). Each treatment n=18. See Table A2 for details on the metabolites.

**Figure 6:**
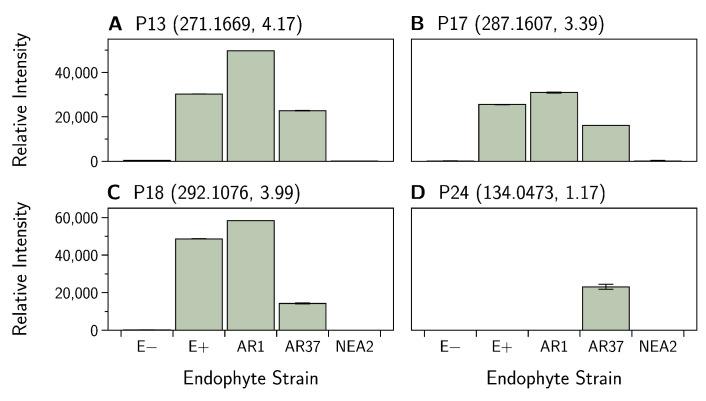
*Epichloë festucae* var. *lolii* endophyte infected *Lolium perenne* root exudate metabolites captured in positive ion mode. Bar graphs showing variation in the relative intensity of metabolites (means ± SEM). See Table A2 for (**A**) P13, (**B**) P17, (**C**) P18, and (**D**) P24 metabolite details. Numbers in parentheses denote the: (mass, retention time).

Hierarchical clustering of the data showed that endophyte treatments separate into two groups, with E− and NEA2 forming one group and E+, AR1, and AR37 forming the other group (Figure 5). Within the second grouping, AR1 and AR37 are more similar to each other than to E+. The majority of compounds are similar between endophyte treatment groups. However, there are trends worth noting. Within E− and NEA2, there are three compounds P13, (Figure 6A), P17 (Figure 6B), and P18 (Figure 6C) at low-intensity clustering together, while in the other three endophyte treatments these compounds had higher intensities. AR37 had one compound, P24 (Figure 6D) with a much higher intensity than in other endophyte and non-endophyte treatment groups. See Table A2 for metabolite details.

#### 3.4.2. Negative Ion Mode

An ANOVA found that 102 of 115 compounds were different at a corrected *p*-value (FDR; [51]) cut-off of 0.05. A subsequent Tukey’s HSD test showed where the differences lay (Table A4). We applied a fold-change analysis to the 102 compounds with differences. Sixty entities passed a fold-change cut-off of ≥2.0 (Table A5). Overall, 60% of metabolites were upregulated in E+, 49% in NEA2, 46% in AR37, and 26% in AR1 relative to E− plants. Interestingly, 38.5% of metabolites were upregulated in E+ while being downregulated in the novel endophytes. There were no metabolites that were upregulated concurrently across all endophyte treatments relative to E− treatments.

To address question 4, we first reduced the dimensionality of the 60 metabolites by conducting a principal component analysis. We were able to reduce these 60 variables to three principal components (PC) that together explained almost two thirds of the total variation in the 60 metabolites. The PC-loadings are shown in Table A6. We subjected these three PCs to the contrast analysis described in Section 2.7.1. The results are shown in Figure 4. There we see differences in PC-1 and PC-2 between infected and uninfected plants. There was not convincing evidence to reject the null hypothesis for PC-3.

**Figure 7:**
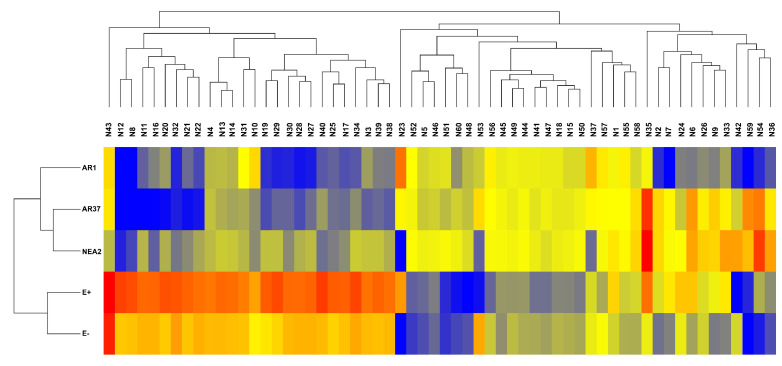
Unsupervised hierarchical clustering of *Lolium perenne* root exudate metabolites, obtained by LC–MS in the negative ion mode. These are the 60 metabolites found to be differentially-expressed (p<0.05 and fold-change >2.0). Metabolites are grouped by fungal endophyte treatments based on similar intensity profiles. Colors represent normalized intensity values (yellow is the center, blue represents low intensity, red represents high intensity). Each treatment n=18. See Table A5 for metabolite details.

**Figure 8:**
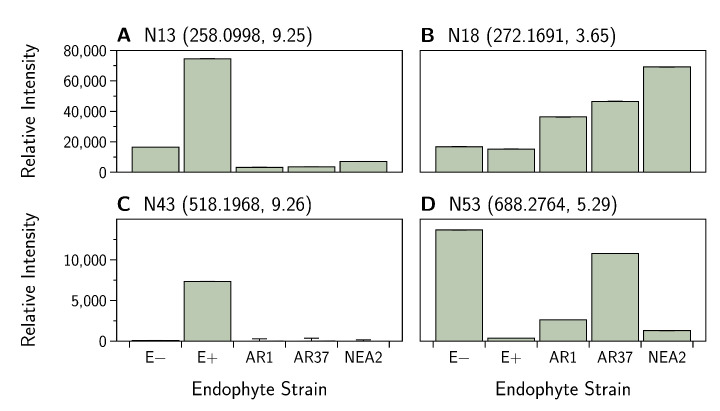
*Epichloë festucae* var. *lolii* endophyte infected *Lolium perenne* root exudate metabolites captured in negative ion mode. Note that the intensities displayed in (**A**) have been divided by 10 to facilitate display. Bar graphs show variation in the relative intensity of metabolites (means ± SEM). See Table A5 for (**A**) N13, (**B**) N18, (**C**) N43 and (**D**) N53 metabolite details. Numbers in parentheses denote the: (mass, retention time).

Hierarchical clustering of the data shows that endophyte treatments separate into two groups with E− and E+ forming the first group and the novel endophytes (AR1, AR37, and NEA2) forming the second group (Figure 7). Within the second grouping, AR37 and NEA2 are more similar to each other than to AR1. For the first 26 compounds in the hierarchical clustering map, there is a general trend of higher intensities for the E+ and E− treatments and lower intensities for the novel endophyte treatments. However, the E+ treatment consistently had higher intensities than the E− treatment. Where a compound was present in all plants, it was most common for E+ to have the highest relative intensity (as an example N13; Figure 8A); however, there were notable exceptions for E− (N53; Figure 8B) and NEA2 (N18; Figure 8C). One additional compound of interest was in E+ (N43; Figure 8D). Here the compound was dominant in E+ presenting in all 18 replicates while showing low relative abundance in E− presenting in 11 replicates, and virtual absent from the other treatments (AR1 n=5, AR37 n=6, NEA2 n=3).

### 3.5. Do the Exudate Metabolomes Differ between Plants Infected with Different Strains?

For these analyses we excluded the E− plants and conducted the ANOVA and Tukey’s HSD test as described in Section 2.7.2.

#### 3.5.1. Positive Ion Mode

**Figure 9:**
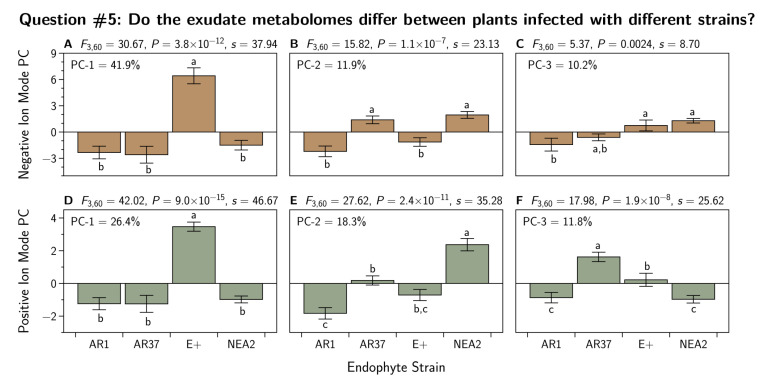
Principal component analysis (PCA) performed on the metabolomic profiles of *Lolium perenne* root exudates infected with strains of *Epichloë festucae* var. *lolii* endophyte. The negative ion PCA is based on 60 metabolites (**A**–**C**) and the positive ion PCA is 24 metabolites (**D**–**F**) found to be differentially-expressed in at least one of the endophyte treatments, with p<0.05 and fold-change >2.0. Each treatment n=18. Plotted are the means and standard errors of the means. Additionally shown are the ANOVA, Shannon transformation indices (*s*), and Tukey’s HSD test results for each principal component (PC). For the HSD test, strains with different letters have different mean values. Percentages in each subfigure represent the percentage of the total variation in the metabolite set that is explained by each PC.

We again used PCA to reduce the dimensionality of the 24 metabolites to three independent principal components (see Table A3 for loadings). The results are shown in Figure 9. Here we see large differences between the strains for all three principal components.

#### 3.5.2. Negative Ion Mode

We again used PCA to reduce the dimensionality of the 60 metabolites to three independent principal components (see Table A6) as described in Section 2.7.2. The results are shown in Figure 9. Here we see large differences between the strains for all three principal components.

### 3.6. Do Relative Abundances of Any of the Exudate Metabolites Correlate with Endophyte Concentration?

We excluded the E− plants to examine the relationship between exudate metabolites and our estimate of endophyte concentration. We calculated Spearman’s ρ to examine the correlation between endophyte concentration and each individual metabolite in both ionization modes. The results are shown in the Supplemental Material. Although some of these ρ seemed important, none survived the FDR correction. It is also worth noting that all of the correlations were relatively weak, falling between −0.35 and +0.35.

## 4. Discussion

The presence of *E. festucae* var. *lolii* in *L. perenne* alters grass physiology, including morphology and phytochemistry, which can have multitrophic implications in the above-ground ecosystem. How changes in *L. perenne* morphology and phytochemistry influence the below-ground ecosystem remains unclear. Identification of changes in root exudate chemistry of fungal endophyte-infected grasses will facilitate our understanding of plant–fungal endophyte contributions to the soil ecosystem and their potential ecological outcomes. We begin the discussion by reexamining the questions we enumerated in the introduction.

### 4.1. Does Plant ‘Performance’ Differ between Infected and Uninfected Plants?

We found that endophyte-infected plants produced more biomass than uninfected plants (Figure 1). This is not surprising since the growth benefits of infection have been previously reported and is the basis of claims for ‘endophyte-enhancement’ in both the forage and turfgrass industries.

### 4.2. Does Plant ‘Performance’ Differ between Plants Infected with Different Endophyte Strains?

We found differences between plants infected with different endophyte strains (Figure 2). We found that NEA2 and AR37 infected plants performed better than E+ or AR1 infected plants. Nevertheless, Geddes-McAlister et al. [55] used the same *L. perenne* cultivar (Alto) and the same endophyte strains as we did and they did not find strain specific effects on biomass production. Other experimental details may explain this discrepancy, but minimally these differences suggest there is much still to understand about this plant–fungal interaction.

### 4.3. Do the in Planta Endophyte Concentrations Differ between Endophyte Strains?

We found that NEA2 infected plants had lower endophyte concentrations than E+ or AR37 plants (AR1 had intermediate concentrations; see Figure 3). These results are very similar to those found by Geddes-McAlister et al. [55].

### 4.4. Do the Exudate Metabolomes Differ between Infected and Uninfected Plants?

We found large differences in the metabolomes of infected and uninfected plants for two out of three principal components in either ion mode (Figure 4). For metabolites detected in the positive ion mode, E− and NEA2 infected plants are more similar to each other than they are to plants infected with the other three endophyte strains (Figure 5). For metabolites detected in the negative ion mode, E+ and E− plants were more similar to each other than they were to the novel endophyte strains (Figure 7).

### 4.5. Do the Exudate Metabolomes Differ between Plants Infected with Different Strains?

We found large differences among exudate metabolomes from the different endophyte strains for all principal components (Figure 9). This is consistent with such differences found in the plant tissue metabolome by Geddes-McAlister et al. [55] although the exudate contained far fewer metabolites than the plant tissue.

### 4.6. Do Relative Abundances of Any of the Exudate Metabolites Correlate with Endophyte Concentration?

We did not find convincing evidence for important correlations between the endophyte concentration and the relative abundances of any of the individual metabolites (Figure S1).

### 4.7. General Discussion

#### 4.7.1. Endophyte Infection

We found that the concentration of endophyte infection (copies ng^−1^ gDNA) of plant sheath tissue was dependent on endophyte strain. In five of 67 sheath tissue samples, endophyte concentration was below the detection limit (AR1 =1, AR37 =1, and NEA2 =3). E+ and AR37 had the highest concentrations (Figure 3) and were not different from one another. E+ and AR37 had 34% higher concentrations than AR1 and 69% higher concentrations than NEA2. AR1 had a 27% higher concentration than NEA2. Different levels of endophyte concentration between strains is consistent with the literature. However, rather than being of equal concentrations as in this current study, others have found the E+ strain to have higher concentrations than AR37 [10,44]. While Tian et al. [56] found E+, AR37, and NEA2 to have similar endophyte concentrations, which were all higher than the AR1 strain. In contrast to this study, where NEA2 had the lowest levels of endophyte infection. Different cultivars of *L. perenne* were used in each of these studies; but see Geddes-McAlister et al. [55]. Interestingly, Ryan et al. [44] found that cultivar explained less than 2% of the variability in endophyte concentration, while host plant genotype explained nearly half of the variability, and endophyte strain almost a third. There are most likely several factors contributing to variability in endophyte infection levels, including within plant colonization success from one tiller to another; environmental factors such as light and temperature; and host-endophyte genetics [44,55,56,57].

#### 4.7.2. Biomass

The fungal endophyte strain NEA2 outperformed all other endophyte strains, producing more root and shoot biomass on average. This result is similar to Tian et al. [56], who found that the root and leaf blade dry weight for NEA2 was greater than that for E+ or AR37. However, they also found NEA6 and E− plants to have more biomass than E+ and AR37, which was not the case for E− in our current study. Several field studies have demonstrated that perennial ryegrass infected with novel endophyte strains provide improvements in pasture productivity and persistence compared to E− plants [58,59]. Additionally, field studies have found that novel endophyte strains can provide comparable improvements to each other in terms of yield [60]. However, some studies have found that novel endophyte strains can outcompete each other. For example, AR37 resulted in higher yields than E+ and AR1 [61], and another where AR37 was higher than AR1 [62]. In a glasshouse study, Ryan et al. [44] found a cultivar by endophyte interaction on blade biomass attributed to one AR37 cultivar having higher shoot biomass than some, but not all, of the other AR37 and E+ cultivars. In contrast E+ plants produced less shoot biomass than E−, AR1, and AR37 [63]. Both Bell et al. [63] and the current study considered only one grass cultivar but still observed strain-specific effects in plant growth. Further research focusing on these grass’s genetic components–fungal endophyte strain-specific differences would perhaps elucidate the mechanisms involved.

#### 4.7.3. Metabolites in Root Exudate

Endophyte strain and plant host genotype have been found to influence the synthesis of fungal-derived alkaloids [56,64]. For example, AR37 is one novel strain that synthesizes epoxy-janthitrems, an alkaloid not so far found in other endophyte strains. We also found a single metabolite that seems to be unique to AR 37: *m*/*z* 135.0546 RT 1.17 (P1). However, it is unlikely that this is an epoxy-janthitrem as these fall in the *m*/*z* of 646.3726 to 714.4329 [65]. It is possible that our unidentified compound is a precursor in the epoxy-janthitrem biosynthesis. Regardless, it stands to reason that if alkaloid quality and quantity vary between strains, then other metabolites may be changing between strains.

In the positive ionization mode, the metabolomic profiles for E− and NEA2 treatments were more similar, and E+, AR1, and AR37 treatments were similar. In the negative ionization mode, E− and E+ were more similar to each other, and AR1, AR37, and NEA2 were more similar to each other. This is in contrast to Wakelin et al. [16], who found that endophyte-infected samples separated out from uninfected samples in *L. perenne* (cv. Samson). They also found that root exudate chemistry differed between the two novel endophyte strains AR1 and AR37. Although the metabolomic profiles did not separate out the E− from the endophyte infected plant exudates in our study, there was a substantial number of individual metabolites that varied between the endophyte treatments and that were different from E− plants. Endophyte treatment groups have also been found to have distinct metabolite patterns in above-ground tissues [7,66,67]. Wakelin et al. [16] found alkane-type (lipid) compounds to be the most variable between the endophyte treatments. In the current study, almost every compound class in both positive and negative ion modes had a high degree of variability between endophyte treatments compared to E−. Although the Wakelin et al. [16] study utilized *L. perenne* infected with AR1 or AR37 fungal endophyte strains or left uninfected (E−), their study differs in plant cultivar, growth and extraction medium (soil), and analytical technique (GC–MS). There is an overlap in the chemical classes that can be captured with GC–MS and LC–MS, but certain chemical classes are most compatible with one technique or the other. Guo et al. [14] conducted a comprehensive root exudate study using *Schedonorus arundinaceus* and *E. coenophiala*. They also utilized GC–MS and found that both endophyte strain and plant cultivar influenced the composition of root exudates. Most notably were the differences in some lipids, phenolics, amines, and sugars between the endophyte strains. Although we did not capture sugars commonly observed in root exudates in the current study, the pattern of endophyte strain-specific effects on root exudate composition found in Guo et al. [14] were similar to this study.

The study of root exudate metabolites in grass–fungal endophyte associations is rare. It was therefore difficult to make clear connections between the compounds we observed with those of other studies. Improvements in the root exudation collection and preparation for LC–MS could be made. Root exudates were measured directly from deionized water, with no concentration of the samples or use of an extraction solvent. Sampling of the root exudates directly from the water most likely had a strong dilution effect, resulting in lower peak intensities and potentially the loss of metabolites during recursive molecular feature extraction. The use of multiple extraction solvents to separate polar and non-polar fractions, and protein fractions of the root exudate, would allow for multiple different LC–MS analyses to capture a broader range of metabolites.

### 4.8. Conclusions

In this study, we were interested in identifying changes in the plant exudate metabolome due to fungal endophyte infection, i.e., the “plant–fungal exudate metabolome.” We focused specifically on root exudate composition changes as very few studies have been conducted on root exudate chemistry in plant–fungal endophyte associations. We found that the presence of fungal endophytes in *L. perenne* affected plant growth and root exudate chemical composition and that these effects were related to the fungal strain. The isolation of an unknown metabolite (P1, *m*/*z* 135.0546 RT 1.17) unique to the AR37 endophyte strain suggests that there are more compounds yet to discover in these associations that may provide mechanistic explanations for fungal endophyte’s fitness benefits to their plant hosts. This study demonstrates the complexity of the plant–fungal association and the fungal endophyte’s role in altering plant host chemistry beyond alkaloids. Our results justify further research in this area, and warrant more targeted metabolomic approaches, paired with other omics techniques and NMR. Such targeted methods will aid in compound identification and mapping of metabolomic pathways to connect the chemistry with the biology. 

## Data Availability

Patchett, Aurora, 2021, Lperenne_Root_Exudate_Metabolomic_Profiles, doi.org/10.7910/DVN/MECRW6, Harvard Dataverse, V1.

## Supplementary Materials

The following is available online at https://www.mdpi.com/2309-608X/7/2/148/s1. Supplementary Figure S1 Spearman’s ρ for metabolite correlations with endophyte concentrations.

## Appendix A. Metabolite Details

**Table A1:** Number of metabolites detected in positive ionization mode that are differentially expressed (blue) or not differentially expressed (green) between treatment groups based on a Tukey’s HSD Post Hoc test (p<0.05).

Treatment	E−	E+	AR1	AR37	NEA2
E−	41	12	8	15	9
E+	29	41	20	19	26
AR1	33	21	41	5	10
AR37	26	22	36	41	4
NEA2	32	15	31	37	41

**Table A2:** Differentially expressed root exudate metabolites detected in positive ionization mode from *Lolium perenne* plants infected with strains of *Epichloë festucae* var. *lolii* (E+, AR1, AR37, and NEA2; each treatment with n=18). Fold-change data are based on the abundance difference between uninfected (E−) plants and individual treatment groups. |Fold-change|≥2 represent downregulated (negative, red) or upregulated (positive, blue) metabolites relative to endophyte free plants. Values in black show either downregulated (negative value) or upregulated (positive value) metabolites that did not pass the fold-change filter. For ease of presentation, the fold-changes are expressed as ${\mathrm{Log}}_{2}\left(fold-change\right)$. The *s*-value denotes the Shannon information transformation of the *p*-value, see Section 2.7.

Positive Ionization Mode						Fold-Change (Log_2_)			
Mass (Code)	*m*/*z*	RT	Fragments	P(1)	*p*-Value (*s*-Value)	E+	AR1	AR37	NEA2
134.0474 (P1)	135.0546	1.17	53.0380, 56.9632, 80.0493, 107.0589, 135.0548	−0.220	$7.50\times 10^{-19}$ (60.21)	−0.09	−1.29	11.40	−1.56
151.0984 (P2)	152.0967	1.74	65.1095, 67.0532, 68.9968, 92.0562, 95.0479	0.043	$9.13\times 10^{-3}$ (6.77)	1.36	−1.26	1.68	0.52
218.0929 (P3)	219.0988	7.15	91.0534, 117.0314, 121.0629, 145.0618, 187.0733	0.118	$9.13\times 10^{-3}$ (6.77)	3.87	1.63	5.24	5.24
220.1085 (P4)	221.1179	7.02	95.0503, 162.8361, 177.1276	0.128	$1.62\times 10^{-3}$ (9.27)	6.19	1.58	5.43	7.18
222.1232 (P5)	223.1362	7.01	61.9898, 178.1371, 208.9768, 223.1332, 707.5326	0.185	$1.55\times 10^{-5}$ (15.98)	2.04	−1.97	−3.01	−1.34
222.1232 (P6)	223.12959	6.97	79.0533, 91.0534, 93.0692, 149.0943, 150.1018	0.337	$6.44\times 10^{-10}$ (30.53)	1.81	−7.66	−5.33	−2.40
224.1392 (P7)	225.1462	7.40	79.0528, 91.0534, 105.0685, 119.0836, 133.0993	0.361	$2.29\times 10^{-6}$ (18.73)	2.06	−5.25	−5.38	−1.74
241.1657 (P8)	242.1716	7.47	79.0523, 99.0434, 133.1000, 147.1141, 207.1337	0.181	$6.56\times 10^{-8}$ (23.86)	1.36	−2.49	−3.94	−2.07
256.1311 (P9)	257.1365	5.70	105.0683, 119.0838, 123.0781, 165.0887, 175.1106	0.146	$2.98\times 10^{-7}$ (21.68)	2.31	−0.93	−4.97	−4.87
258.1919 (P10)	259.2001	4.21	55.0537, 59.0120, 83.0843, 100.1112, 115.0749	−0.039	$1.97\times 10^{-3}$ (8.99)	−1.37	0.93	1.16	1.49
271.1669 (P11)	272.1741	4.16	97.0751, 125.1066, 131.0482, 168.1123	−0.379	$1.61\times 10^{-11}$ (35.86)	5.95	6.85	5.53	−2.84
275.1267 (P12)	276.1330	9.27	43.52241, 57.0700212, 61.836, 75.67711, 81.5744, 149.02072, 159.1144, 160.5581, 171.1357, 177.1268	0.149	$2.17\times 10^{-13}$ (42.07)	1.77	−2.92	−2.74	−1.94
277.124 (P13)		9.26		0.263	$1.97\times 10^{-8}$ (25.60)	1.79	−5.37	−4.79	−2.31
287.1607 (P14)	288.1695	3.34	58.06496, 125.1066, 147.04361, 168.1123, 241.5898, 288.1684	−0.376	$1.21\times 10^{-13}$ (42.92)	7.60	8.06	6.93	−0.91
292.1076 (P15)	293.1167	4.01	219.1086, 234.1209, 245.1129, 293.1163	−0.424	$4.62\times 10^{-16}$ (50.94)	7.40	7.84	5.62	−4.28
332.1135 (P16)	333.1155	5.24	289.0938, 317.0889, 333.1201	0.052	$4.84\times 10^{-2}$ (4.37)	0.34	−1.38	0.46	−0.35
353.2758 (P17)	354.2816	10.72	45.0329, 57.0692, 59.0483, 87.0431, 103.0738	0.013	$4.08\times 10^{-4}$ (11.26)	−2.28	0.17	−0.81	0.15
358.2297 (P18)	359.2363	10.83	99.0435, 129.0881, 359.2350	0.019	$1.80\times 10^{-3}$ (9.12)	−2.40	0.24	−0.85	0.21
371.1752 (P19)	372.1870	4.49	45.0339, 59.0498, 89.0607, 103.0395, 147.0663	0.088	$3.79\times 10^{-8}$ (24.65)	4.07	1.45	8.36	8.28
381.3048 (P20)		10.82		0.004	$2.29\times 10^{-6}$ (18.73)	−5.46	0.17	−0.67	0.35
387.2441 (P21)	388.2516	4.54	45.0334, 57.0699, 89.0595, 133.0855, 149.0223	0.029	$5.89\times 10^{-4}$ (10.73)	0.48	0.37	1.48	1.74
424.3398 (P22)		5.36		−0.075	$2.44\times 10^{-2}$ (5.36)	0.41	3.27	2.52	3.35
433.205 (P23)	434.2121	5.06	124.1104, 288.1565, 434.2117	0.089	$1.68\times 10^{-3}$ (9.22)	−3.53	−2.39	0.92	2.23
434.3239 (P24)	435.3262	5.47	57.0680, 109.1018, 118.0844, 151.0729, 176.1059, 207.0601, 211.1391, 298.0424, 421.1824	−0.069	$4.84\times 10^{-2}$ (4.37)	−0.33	2.36	1.74	2.55

**Table A3:** Positive ion mode loading matrix. Heavy loadings (>0.5 or <−0.5) are highlighted. See Table A2 for details on the individual metabolites.

	Positive Ion with E−			Positive Ion without E−		
Metabolite	PC-1	PC-2	PC-3	PC-1	PC-2	PC-3
P1	−0.23	0.02	0.47	−0.15	0.09	0.58
P2	0.23	0.26	0.62	0.23	0.38	0.57
P3	0.12	0.48	0.45	0.17	0.63	0.24
P4	0.14	0.39	0.45	0.20	0.60	0.14
P5	0.85	0.04	−0.05	0.83	0.10	−0.24
P6	0.79	0.12	−0.05	0.75	0.26	−0.10
P7	0.85	0.14	−0.09	0.78	0.25	−0.27
P8	0.86	0.01	−0.21	0.83	0.11	−0.33
P9	0.68	−0.24	−0.16	0.65	−0.25	−0.21
P10	−0.55	0.03	0.00	−0.58	−0.01	−0.08
P11	−0.04	−0.77	0.24	0.21	−0.76	0.26
P12	0.95	−0.02	−0.04	0.93	0.10	−0.11
P13	0.81	0.01	−0.06	0.77	0.14	−0.14
P14	−0.10	−0.72	0.35	0.20	−0.72	0.34
P15	−0.02	−0.81	0.21	0.23	−0.79	0.26
P16	0.38	0.33	0.36	0.34	0.40	0.43
P17	−0.26	0.43	−0.62	−0.42	0.29	−0.60
P18	−0.25	0.44	−0.63	−0.37	0.29	−0.63
P19	−0.07	0.53	0.58	−0.04	0.65	0.40
P20	−0.37	0.42	−0.56	−0.51	0.25	−0.51
P21	−0.06	0.61	0.56	−0.09	0.70	0.38
P22	−0.48	0.00	0.16	−0.52	−0.02	0.06
P23	−0.17	0.49	0.13	−0.32	0.48	0.10
P24	−0.45	−0.09	0.14	−0.49	−0.05	0.03

**Table A4:** Number of metabolites detected in negative ionization mode that are differentially expressed (blue) or not deferentially expressed (green) between treatment groups based on a Tukey’s HSD Post Hoc test (p<0.05).

Treatment	E−	E+	AR1	AR37	NEA2
E−	102	41	27	35	34
E+	61	102	78	73	76
AR1	75	24	102	8	20
AR37	67	29	94	102	5
NEA2	68	26	82	97	102

**Table A5:** Differentially expressed root exudate metabolites detected in negative ionization mode from *Lolium perenne* plants infected with strains of *Epichloë festucae* var. *lolii* (E+, AR1, AR37, and NEA2; each treatment with n=18). Fold-change data are based on the abundance difference between uninfected (E−) plants and individual treatment groups. |Fold-change|≥2 represent downregulated (negative, red) or upregulated (positive, blue) metabolites relative to endophyte free plants. Values in black show either downregulated (negative value) or upregulated (positive value) metabolites that did not pass the fold-change filter. For ease of presentation, the fold-changes are expressed as ${\mathrm{Log}}_{2}\left(fold-change\right)$. The *s*-value denotes the Shannon information transformation of the *p*-value, see Section 2.7.

Negative Ionization Mode						Fold-Change (Log_2_)			
Mass (Code)	*m*/*z*	RT	Fragments	P(1)	*p*-Value (*s*-Value)	E+	AR1	AR37	NEA2
183.0886 (N1)	182.0839	5.06	22.38964, 24.00521, 28.29697, 28.74668, 44.33648, 61.9898, 112.03114, 128.2595	0.019	$2.69\times 10^{-2}$ (5.22)	1.60	0.64	0.84	1.23
222.0293 (N2)	221.0305	5.75	79.9590, 157.0675, 221.0303	0.030	$1.89\times 10^{-4}$ (12.37)	1.99	−1.23	3.33	3.31
222.1244 (N3)	221.1179	7.02	27.59896, 29.39881, 35.02124, 38.24089, 41.62328, 95.05037, 162.8361, 177.1276	0.158	$2.97\times 10^{-7}$ (21.68)	1.32	−3.01	−4.16	−2.31
224.1391 (N4)	223.1362	7.01	20.83417, 21.21704, 26.55221, 31.06935, 42.66667, 61.98988, 178.1371, 208.9769, 223.13328	0.105	$2.14\times 10^{-15}$ (48.73)	1.24	−2.53	−2.38	−2.50
226.1654 (N5)	225.1462	7.40	79.0528, 91.05348, 105.068589, 119.0837, 133.09936	−0.100	$1.45\times 10^{-2}$ (6.11)	0.50	2.44	2.23	2.94
236.1024 (N6)	235.0976	7.15	62.00181, 78.683, 80.7794, 84.2328, 105.03409, 158.0746, 157.5531, 177.0557, 235.0948	0.046	$1.39\times 10^{-3}$ (9.49)	2.73	−0.75	3.54	3.32
240.134 (N7)		6.19		0.052	$1.47\times 10^{-3}$ (9.41)	2.74	−2.50	2.46	2.21
240.1347 (N8)	239.1303	6.24	45.88506, 51.70296, 57.0356, 62.9934, 66.41873, 85.09677, 86.6111, 107.05075, 123.08235, 239.1305	0.233	$1.47\times 10^{-3}$ (9.41)	2.74	−2.50	2.46	2.21
252.0967 (N9)	251.0942	5.86	57.03481, 75.0168, 80.31151, 82.07862, 105.03536, 157.8586, 173.8734, 190.06535, 221.08245	−0.005	$1.20\times 10^{-2}$ (6.38)	2.13	0.11	3.34	3.19
256.0851 (N10)	255.0256	7.05	38.37795, 41.39448, 47.78295, 61.02047, 77.82369, 79.9573, 83.2637, 129.09133, 143.05869	0.077	$2.52\times 10^{-14}$ (45.17)	1.45	0.44	−2.33	−2.37
256.1289 (N11)	255.1250	5.75	125.0986, 255.1237	0.224	$9.81\times 10^{-9}$ (26.60)	1.39	−4.23	−7.36	−2.70
258.0985 (N12)	257.0962	8.49	19.59993, 21.10193, 22.20991, 52.4277, 28.36111, 35.59788, 145.31803, 178.1321, 221.1178	0.118	$9.81\times 10^{-9}$ (26.60)	2.46	−6.03	−6.49	−5.03
258.0998 (N13)		9.25		0.118	$2.14\times 10^{-15}$ (48.73)	1.46	−2.50	−2.76	−2.24
260.0974 (N14)		9.25		0.120	$4.39\times 10^{-15}$ (47.70)	1.48	−2.55	−2.84	−2.22
262.1423 (N15)	261.1404	3.64	18.2351, 19.01389, 24.10197, 27.78419, 32.2279, 197.9015	−0.064	$3.65\times 10^{-3}$ (8.10)	−0.76	0.81	1.03	1.23
270.1086 (N16)	269.1059	5.25	46.27977, 55.020019, 65.97169, 98.9411, 105.3432, 135.08309, 163.1151, 181.1243, 208.1078, 228.3399	0.228	$8.04\times 10^{-10}$ (30.21)	1.45	−3.63	−7.85	−4.29
272.1238 (N17)	271.1209	5.10	61.86755, 75.93481, 120.3023, 165.1305, 180.5423, 207.1042, 209.1204, 290.1051, 227.1302, 271.1204	0.175	$9.44\times 10^{-7}$ (20.02)	1.79	−4.32	−3.81	−3.20
272.1691 (N18)	271.1663	3.61	29.33423, 31.84118, 36.40309, 44.9984, 53.87695, 83.04982, 186.2128	−0.065	$1.92\times 10^{-3}$ (9.03)	−0.85	0.98	0.91	1.01
274.0957 (N19)	273.0923	7.52	79.9571, 120.9018, 239.0403	0.185	$1.46\times 10^{-7}$ (22.71)	2.50	−4.28	−3.74	−1.61
278.1108 (N20)		5.72		0.193	$5.91\times 10^{-8}$ (24.01)	2.24	−2.78	−5.46	−2.42
282.1448 (N21)	281.1423	6.33	28.68056, 37.10545, 44.9993, 47.4394, 49.23808, 71.07635, 125.9432, 237.1522, 238.1544, 281.1406	0.175	$1.12\times 10^{-7}$ (23.09)	1.82	−3.75	−5.46	−3.17
290.1111 (N22)		7.00		0.194	$1.10\times 10^{-8}$ (26.44)	1.16	−4.75	−5.71	−3.03
292.1089 (N23)	291.1030	4.00	55.02972, 74.5326, 110.9243, 171.9102, 231.09958, 257.1145, 291.1018	0.004	$6.63\times 10^{-15}$ (47.10)	7.23	7.98	5.57	−1.22
294.1081 (N24)	293.1051	6.77	106.0435, 135.1298, 165.0555, 172.09136, 196.4521, 201.8361, 221.0823, 231.1034, 242.0645	0.075	$2.67\times 10^{-5}$ (15.19)	1.41	−1.97	−0.64	0.40
296.0738 (N25)		9.25		0.177	$1.30\times 10^{-7}$ (22.88)	2.14	−3.69	−3.43	−3.20
302.1238 (N26)	301.1220	4.97	45.8685, 57.44, 57.51875, 117.0584, 160.0413, 161.0501, 283.1125	0.077	$3.59\times 10^{-2}$ (4.80)	0.20	−1.30	1.17	1.74
316.0583 (N27)		9.25		0.179	$4.01\times 10^{-10}$ (31.21)	1.46	−5.54	−4.04	−2.60
316.106 (N28)	315.1013	7.09	32.54166, 61.9881, 66.41003, 67.7094, 90.87622, 146.9665, 158.7408	0.174	$8.04\times 10^{-10}$ (30.21)	1.29	−5.70	−4.67	−2.81
316.106 (N29)		8.19		0.189	$4.57\times 10^{-8}$ (24.38)	2.57	−4.84	−3.57	−1.93
318.1037 (N30)	317.1014	7.12	61.9893, 121.0290, 278.2160, 299.5229	0.189	$7.25\times 10^{-8}$ (23.72)	1.41	−5.30	−4.10	−3.45
326.0872 (N31)		9.26		0.092	$3.98\times 10^{-15}$ (47.84)	1.25	−1.09	−2.65	−2.44
328.0832 (N32)		8.54		0.190	$5.53\times 10^{-11}$ (34.08)	1.33	−5.75	−5.87	−4.26
338.1705 (N33)	337.1666	7.89	59.19598, 62.61855, 89.14405, 106.0433, 150.03368, 165.05589, 231.7807, 317.8932, 337.16603	0.022	$2.22\times 10^{-3}$ (8.82)	2.69	0.63	2.57	4.07
343.0776 (N34)		9.25		0.177	$2.84\times 10^{-9}$ (28.39)	1.82	−4.62	−3.68	−2.55
347.1702 (N35)	346.1657	8.55	37.0372, 42.8426, 58.7676, 61.9881, 67.0801, 73.8238, 133.5098	0.013	$5.18\times 10^{-5}$ (14.24)	2.94	0.15	4.08	7.17
352.1333 (N36)	351.1321	3.59	57.0366, 59.01548, 83.1737, 91.31488, 98.45027, 99.0455, 101.0255, 104.3767, 194.56109, 337.0621	−0.044	$2.73\times 10^{-2}$ (5.19)	1.16	0.16	3.66	4.95
362.1447 (N37)	361.1418	4.21	29.44697, 35.12963, 37.57853, 46.3367, 60.61403, 90.72506, 160.7155, 174.05642, 269.1017	0.008	$2.03\times 10^{-5}$ (15.59)	−0.34	1.72	0.50	−2.33
418.9382 (N38)	417.9399	9.26	160.8439, 162.8406, 417.9403	0.164	$9.81\times 10^{-9}$ (26.60)	1.30	−3.70	−4.30	−2.76
420.9378 (N39)	419.9326	8.57	34.55704, 37.63, 40.03553, 43.15385, 59.46324, 189.9559, 333.1031, 346.0412	0.152	$4.57\times 10^{-9}$ (27.71)	1.70	−3.49	−3.88	−2.22
422.9337 (N40)		9.25		0.170	$7.74\times 10^{-9}$ (26.95)	2.31	−4.30	−3.04	−3.88
452.3299 (N41)		5.36		−0.075	$1.10\times 10^{-2}$ (6.50)				
479.212 (N42)	478.2061	5.10	25.98425, 27.50015, 42.875, 44.99806, 80.4495, 115.0385, 286.1415, 315.4149, 401.1564	−0.067	$1.32\times 10^{-3}$ (9.57)	−4.26	−3.06	−0.07	2.51
518.1987 (N43)		9.26		0.240	$7.05\times 10^{-15}$ (47.01)	6.50	−3.40	−3.60	−5.34
550.3019 (N44)	549.2983	5.39	78.9593, 96.9619, 98.9566, 549.3004	−0.064	$4.15\times 10^{-3}$ (7.91)	−0.38	1.18	1.34	1.41
556.2976 (N45)		5.36		−0.071	$7.49\times 10^{-3}$ (7.06)	0.17	1.68	1.83	1.90
558.2961 (N46)		5.36		−0.087	$3.59\times 10^{-2}$ (4.80)	0.38	2.14	1.86	2.51
573.2877 (N47)		5.36		−0.074	$1.70\times 10^{-2}$ (5.88)	−0.90	1.32	1.44	1.11
575.2794 (N48)		5.36		−0.142	$6.77\times 10^{-3}$ (7.21)	−1.98	1.93	2.00	2.51
583.3164 (N49)		5.36		−0.059	$1.01\times 10^{-3}$ (9.95)	−0.67	1.01	0.90	1.05
600.3065 (N50)		5.36		−0.070	$1.58\times 10^{-2}$ (5.99)	−0.82	0.93	1.33	1.49
651.3033 (N51)		5.36		−0.132	$7.07\times 10^{-3}$ (7.14)	−0.18	3.19	2.48	3.59
685.2842 (N52)		5.36		−0.105	$1.49\times 10^{-4}$ (12.72)	0.69	3.58	3.39	3.51
688.2764 (N53)	687.2796	5.27	205.8351, 239.5135, 281.09199, 466.2721, 581.21507, 653.9155, 687.2787	−0.005	$3.79\times 10^{-5}$ (14.69)	−5.95	−2.52	−0.91	−4.44
768.1591 (N54)	767.1577	3.92	82.3149, 553.1354, 603.8227, 767.1552	−0.087	$1.98\times 10^{-7}$ (22.27)	2.69	0.25	6.54	7.71
774.4346 (N55)		6.00		−0.045	$4.51\times 10^{-3}$ (7.79)	0.55	1.77	1.50	1.60
854.4604 (N56)		5.99		−0.074	$2.22\times 10^{-3}$ (8.82)	−1.76	1.29	1.05	0.89
858.483 (N57)		5.99		−0.043	$4.50\times 10^{-3}$ (7.80)	−1.46	0.50	0.16	−0.04
900.5351 (N58)		5.99		−0.039	$4.49\times 10^{-3}$ (7.80)	0.75	0.79	2.12	2.28
999.2011 (N59)	998.2025	3.90	28.9734, 33.5092, 96.9584, 298.4722, 815.2975, 998.2023	−0.099	$4.22\times 10^{-6}$ (17.86)	0.97	−1.07	7.10	6.11
1403.0048 (N60)	1402.0042	5.99	677.4985, 723.5008, 815.1445	−0.137	$3.36\times 10^{-2}$ (4.89)	−0.99	1.36	2.50	3.08

**Table A6:** Negative ion mode loading matrix. Heavy loadings (>0.5 or <−0.5) are highlighted. See Table A5.

	Negative Ion with E−			Negative Ion without E−		
Metabolite	PC-1	PC-2	PC-3	PC-1	PC-2	PC-3
N1	0.33	0.60	−0.14	0.46	0.47	−0.18
N2	0.21	0.78	0.30	0.28	0.81	0.15
N3	0.81	0.00	0.24	0.79	0.04	0.22
N4	0.90	−0.22	0.13	0.88	−0.23	0.17
N5	−0.69	−0.26	0.45	−0.69	−0.17	0.49
N6	0.27	0.76	0.25	0.35	0.74	0.05
N7	0.19	0.41	0.48	0.31	0.46	0.30
N8	0.69	−0.20	0.29	0.68	−0.13	0.34
N9	0.01	0.62	0.34	0.17	0.68	0.11
N10	0.69	−0.31	−0.23	0.68	−0.44	−0.21
N11	0.77	−0.17	0.26	0.74	−0.12	0.30
N12	0.70	−0.21	0.17	0.68	−0.15	0.23
N13	0.96	−0.13	0.15	0.96	−0.14	0.18
N14	0.95	−0.15	0.17	0.94	−0.17	0.20
N15	−0.76	−0.26	0.45	−0.77	−0.09	0.52
N16	0.77	−0.26	0.18	0.75	−0.24	0.21
N17	0.70	−0.09	0.30	0.70	0.03	0.32
N18	−0.80	−0.29	0.41	−0.83	−0.13	0.47
N19	0.74	−0.15	0.36	0.72	−0.11	0.42
N20	0.77	−0.23	0.18	0.76	−0.25	0.23
N21	0.67	−0.14	0.19	0.62	−0.14	0.23
N22	0.77	−0.16	0.23	0.75	−0.11	0.25
N23	0.03	−0.16	−0.29	0.23	−0.41	−0.39
N24	0.67	0.49	0.25	0.68	0.52	0.11
N25	0.79	−0.06	0.22	0.78	−0.06	0.20
N26	0.13	0.64	0.24	0.21	0.71	0.16
N27	0.79	−0.06	0.37	0.77	0.05	0.41
N28	0.71	−0.23	0.34	0.67	−0.15	0.44
N29	0.73	−0.15	0.36	0.75	−0.04	0.41
N30	0.78	−0.01	0.23	0.77	0.06	0.23
N31	0.85	−0.29	−0.07	0.82	−0.36	−0.03
N32	0.72	−0.27	0.27	0.68	−0.20	0.37
N33	0.18	0.72	0.22	0.32	0.76	0.05
N34	0.85	0.00	0.28	0.83	0.04	0.29
N35	0.10	0.64	0.22	0.20	0.64	0.11
N36	−0.08	0.65	0.18	−0.02	0.64	0.01
N37	0.13	−0.02	−0.54	0.10	−0.23	−0.48
N38	0.83	−0.04	0.24	0.82	0.01	0.25
N39	0.78	−0.13	0.32	0.74	−0.09	0.37
N40	0.72	−0.20	0.28	0.68	−0.15	0.35
N41	−0.67	−0.27	0.36	−0.70	−0.16	0.37
N42	−0.14	0.45	−0.05	−0.24	0.48	−0.05
N43	0.72	−0.17	0.23	0.79	−0.17	0.17
N44	−0.73	−0.19	0.43	−0.83	−0.08	0.47
N45	−0.71	−0.16	0.55	−0.82	−0.09	0.52
N46	−0.62	−0.18	0.43	−0.62	−0.04	0.52
N47	−0.63	−0.24	0.33	−0.63	−0.15	0.31
N48	−0.67	−0.17	0.32	−0.69	−0.03	0.32
N49	−0.82	−0.31	0.38	−0.83	−0.15	0.47
N50	−0.64	−0.17	0.51	−0.64	−0.02	0.54
N51	−0.67	−0.28	0.33	−0.67	−0.16	0.47
N52	−0.68	−0.13	0.41	−0.74	−0.07	0.38
N53	0.02	−0.08	−0.32	−0.14	−0.08	−0.29
N54	−0.24	0.70	0.31	−0.16	0.74	0.14
N55	−0.55	0.00	0.41	−0.69	0.01	0.14
N56	−0.56	−0.17	0.11	−0.62	−0.15	0.19
N57	−0.50	−0.15	0.05	−0.56	−0.04	0.14
N58	−0.38	0.30	0.61	−0.25	0.53	0.47
N59	−0.24	0.66	0.23	−0.15	0.76	0.06
N60	−0.66	−0.26	0.46	−0.65	−0.15	0.52
