# Supplementary figures and images for: Comparison of Plant Metabolites in Root Exudates of Lolium perenne Infected with Different Strains of the Fungal Endophyte Epichloë festucae var. lolii
Source: J Fungi (Basel). 2021 Feb 18;7(2):148. doi: 10.3390/jof7020148 (PMC7922862; doi:10.3390/jof7020148)

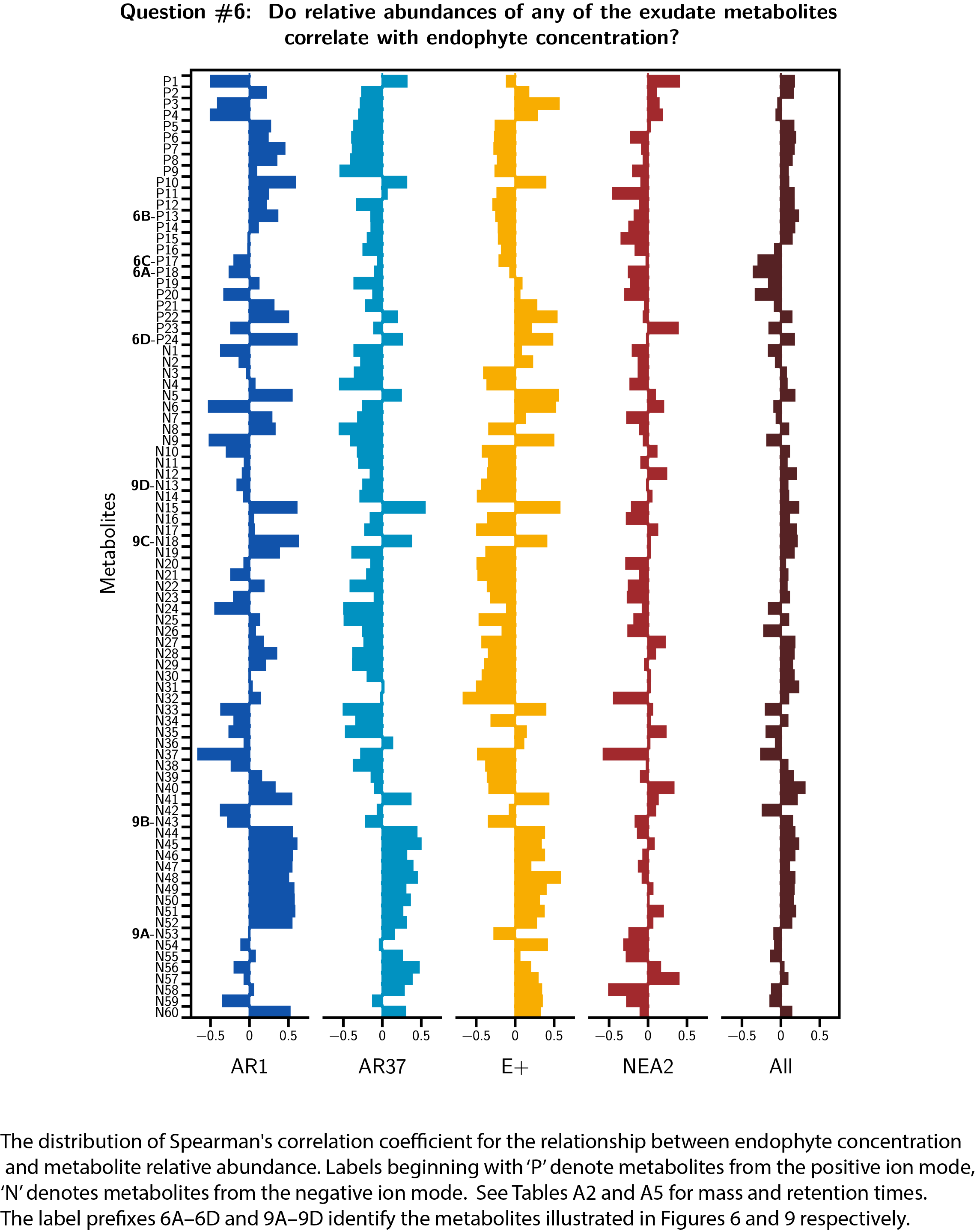

Supplement: Supplementary file 1 [file jof-07-00148-s001.png]
